# Associations between objectively assessed child and parental physical activity: a cross-sectional study of families with 5–6 year old children

**DOI:** 10.1186/1471-2458-14-655

**Published:** 2014-06-27

**Authors:** Russell Jago, Simon J Sebire, Lesley Wood, Laura Pool, Jesmond Zahra, Janice L Thompson, Deborah A Lawlor

**Affiliations:** 1 Centre for Exercise, Nutrition & Health Sciences, School for Policy Studies, University of Bristol, 8 Priory Road, Bristol, BS8 1TZ, UK; 2 School of Sport, Exercise and Rehabilitation Sciences, University of Birmingham, Birmingham B15 2TT, UK; 3 MRC Integrative Epidemiology Unit at the University of Bristol, Oakfield House, Oakfield Grove, Bristol BS8 2BN, UK; 4 School of Social and Community Medicine, University of Bristol, Canynge Hall, Whiteladies Road, Bristol BS8 2PS, UK

**Keywords:** Gender, Parent, Cross-sectional, Exercise

## Abstract

**Background:**

A number of studies have suggested that there is a need to increase the physical activity levels of children. Parents are important influences on children’s behaviour. There is a lack of information about whether there are associations between the physical activity levels of young children and their parents. The current study examined the associations between the physical activity (PA) of parents and their children at age five to six years old, and determined whether any associations differed by child or parent gender or between week and weekend days.

**Methods:**

Cross-sectional study, with 1267 Year 1 pupils (five to six years of age) and at least one parent from 57 primary schools. Children and parents wore an accelerometer for five days and mean minutes of moderate-to-vigorous intensity physical activity (MVPA) per day were derived. We used multivariable linear regression to investigate whether parental and child time spent in MVPA was associated with each other. Each model was adjusted for age, child gender, parent BMI and neighbourhood deprivation with subgroup analysis by child gender.

**Results:**

80% of parents met PA guidelines, however 29% of boys and 47% of girls aged five to six years failed to meet them. Fully-adjusted analyses suggested weak positive associations of parent’s and children’s time spent in MVPA. Every 10 additional minutes of parental MVPA were associated with one additional minute of child MVPA. There was no evidence of a difference in associations for boys and girls or between mothers and fathers.

**Conclusions:**

29% of boys and 47% of girls aged five to six years did not meet PA guidelines indicating that these children would benefit from new approaches that focus on increasing physical activity. There were weak associations between the MVPA of 5–6 year old children and their parents, demonstrating that the time that children are active with their parents is not a major source of physical activity. Clinicians and public health professionals should encourage parents to create opportunities for their children to be active.

## Background

Physical activity (PA) is associated with improved physical and psychological health outcomes [1]. Physical activity levels track from childhood into adulthood [2,3]. A number of studies have reported that large proportions of children do not meet the national guideline of an hour per day of moderate-to-vigorous intensity physical activity (MVPA) [4,5]. PA levels decline with age [6] with the start of primary school being a key transition period [7]. Finding ways to enhance PA at the start of primary school is an important public health target.

Parents are likely to influence children’s PA and could have an important role in increasing their child’s PA [8-10]. Parental influence could be exerted in one of three ways: 1) parent is active with child; 2) parent is active (not necessarily with child) and models active behaviour; and 3) parent facilitates PA for their child [10-12]. There is a lack of information about the association between the PA patterns of parents and their children at the start of primary school. Although previous studies have highlighted that both child and adult PA patterns differ by gender, it is not clear whether there are gender differences in the association between parent and child PA [13,14]. Both children’s and adults’ PA patterns have been shown to differ between weekdays and weekend days [13,15] but whether any association between parental and child PA differs across the week is unknown.

The aim of this study was to examine whether there was evidence of association between the PA of parents and their children at age five to six, and to determine whether associations differed by child or parent gender. A secondary aim was to examine whether associations differed between weekdays and weekend days.

## Methods

We used data from a cross-sectional study (B-ProAct1v) which aimed to identify key factors associated with PA among children in their second year of schooling (known as Year 1 or Y1 in the UK) [16]. Between Jan 2012 and May 2013 250 primary schools, located in Bristol and the surrounding area, were invited to participate in the study. Of the 65 (26%) schools that consented to participate in the study, two withdrew before any children had been recruited and data collection dates could not be scheduled in a further six. All children in Y1 in the remaining 57 schools were eligible to take part.

Children and at least one parent/carer were required to wear an accelerometer for five days. Self-identified first parents completed a questionnaire about personal and family characteristics while second parents provided demographic information only. Written parental consent was obtained for both their own and their child’s participation. The study was approved by the School for Policy Studies ethics committee at the University of Bristol.

**Figure 1:**
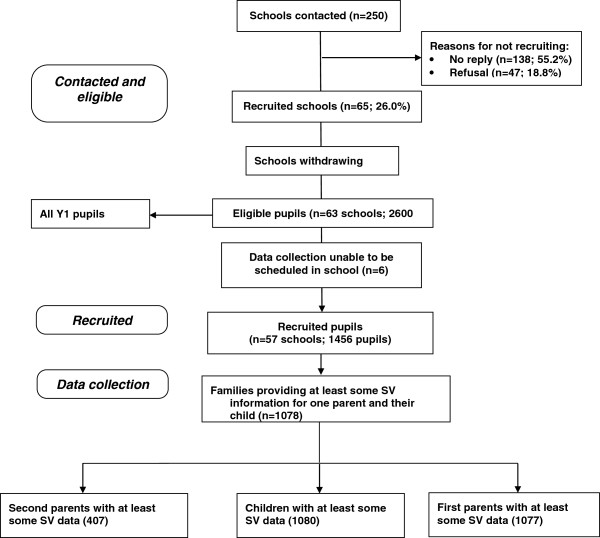
STROBE Study flow of participants.

A total of 57 schools participated in the study with 1456 of a potential 2600 pupils (56%) providing consent. Of the 1456 pupils, 1267 pupils and at least one parent returned an accelerometer. Consistent with the STROBE guidelines, Figure 1 shows the study flow of participants [17].

Children and parents wore an Actigraph GT3X accelerometer for five days including a weekend. Consistent with standard protocols, uniaxial accelerometer data were processed for analysis (see below for details). Parents reported their gender, height and weight, and body mass index (BMI = kg/m^2^) was calculated. Child height was measured to the nearest 0.1 cm using a SECA Leicester stadiometer. Weight was recorded to the nearest 0.1 kg using a SECA 899 digital scale. Body mass index (BMI = kg/m^2^) was calculated and converted to an age and gender specific standard deviation score (BMI z-score) [18,19]. An index of multiple deprivation (IMD) score, using the English Indices of Deprivation (http://data.gov.uk/dataset/index-of-multiple-deprivation), was assigned to each participant based on their home postcode linked to the respective lower layer super output area. A higher IMD score indicates a greater level of deprivation.

### Accelerometer data management

The database was restructured so that parents and associated variables were identified as fathers or mothers rather than as first or second parents. Parents and children were included in the primary analyses if they provided at least three days of valid data where a valid day was defined as the provision of at least 500 minutes of data [20]. Periods of ≥60 minutes of zero values, with an allowance of up to two minutes of interruptions, were defined as accelerometer “non-wear” time and were removed from the analyses [21]. To be included in weekday analysis participants were required to have provided at least two valid days of weekday data; for weekend analysis one valid weekend day was required. We conducted analyses with measured minutes spent in MVPA as a continuous variable which we derived using population specific cut points for children and adults, relating parent PA to that of their child [21,22]. We also categorised participants (parents and children) into two groups based on whether they met UK government guidelines of PA based on minutes of MVPA and then examined the association between whether parents and their children met these guidelines. Adults achieving at least 30 minutes per day were considered to have met the guidelines. For children we used a threshold of 60 minutes of MVPA [23].

### Statistical analysis

Student t-tests and chi-square tests were used to examine differences in the characteristics of participants who provided sufficient accelerometer data and those who were recruited but were excluded due to wearing accelerometers for an inadequate length of time.

We used multivariable linear regression to investigate associations between parental and child MVPA. Each model was adjusted for child gender, parental BMI and age, and household IMD. We subjectively compared the magnitude of associations between gender specific subgroups by examining the point estimates and we also tested statistically for evidence of heterogeneity (difference in magnitude of association between the subgroups) by including the interaction term of parent MVPA*child gender. We used logistic regression analysis, adjusted as described previously, to test whether parent’s meeting the PA guideline predicted children’s meeting of the PA guidelines. Analyses were repeated for weekday and weekend PA. Robust standard errors were used to take account of the clustering of participants within schools. All analyses were conducted in Stata version 12.1 (Statacorp, College Station, TX).

## Results

Summary statistics are given for participants who were included in the fully adjusted models compared with those who were excluded (Table 1). Children who were excluded had significantly higher IMD scores, indicating a greater level of social deprivation (Table 1). Excluded girls but not boys had significantly higher BMI-z scores. Mothers who were excluded had higher IMD scores and were older compared with those who were included whilst there was no difference between the two groups of fathers.

**Table 1:** Characteristics of participants who were included in the overall analysis compared with those who were excluded

	**Included**			**Excluded**			**Difference**		
	**n**	**Mean**	**SD**	**n**	**Mean**	**SD**	**Mean**	**95% CI**	**p**
**Fathers**									
Age (years)	423	40.1	5.4	32	40.6	5.7	0.40	−1.6 to 2.4	0.685
BMI score	423	26.1	3.8	31	26.1	4.3	0.02	−1.4 to 1.4	0.974
IMD score*	423	12.1	9.5	33	14.6	13.4	2.4	−1.1 to 5.6	0.170
**Mothers**									
Age (years)	651	37.5	5.4	63	39.1	6.4	1.5	0.12 to 2.9	0.033
BMI	651	25.0	4.5	66	25.8	4.1	0.84	−0.30 to 2.0	0.150
IMD score*	651	13.6	11.1	73	16.5	13.5	2.9	0.18 to 5.7	0.037
**Children**									
Age (years)	822	6.0	0.42	408	6.0	0.35	0.01	−0.03 to 0.06	0.539
BMI-z score^†^	815	0.20	0.92	399	0.35	0.98	0.14	0.03 to 0.26	0.014
IMD score	822	13.3	10.8	349	19.1	15.9	5.8	4.2 to 7.4	<0.001
**Boys**									
Age (years)	436	6.0	0.41	197	6.0	0.39	0.00	−0.07 to 0.07	0.943
BMI-z score^†^	433	0.26	0.97	189	0.25	0.89	−0.01	−0.17 to 0.15	0.902
IMD score	436	13.7	11.4	178	18.5	15.2	4.8	2.6 to 7.0	<0.001
**Girls**									
Age (years)	386	6.0	0.43	211	6.0	0.32	0.03	−0.04 to 0.09	0.405
BMI-z score^†^	382	0.14	0.86	210	0.43	1.0	0.29	0.14 to 0.45	<0.001
IMD score	386	12.8	10.1	171	19.7	16.7	6.9	4.7 to 9.2	<0.001

*A higher value indicates greater deprivation.

^†^Child BMI was standardized for age.

The average daily time spent in MVPA exceeded the guideline of 30 minutes for mothers and fathers (Table 2), with more than 80% of both sexes reaching this guideline. Just over 70% of boys met the more stringent 60 minutes recommended for children, with an average 72 minutes spent in MVPA. Girls were not as active, with just over half meeting the recommended levels of 60 minutes MVPA per day.

**Table 2:** Physical activity for all participants, and by gender

	**Father**			**Mother**			**Boys**			**Girls**			**All children**		
	**n**	**Mean**	**SD**	**n**	**Mean**	**SD**	**n**	**Mean**	**SD**	**n**	**Mean**	**SD**	**n**	**Mean**	**SD**
MVPA Overall	423	52.1	24.5	651	49.0	22.7	436	72.0	21.2	386	62.4	17.0	822	67.5	19.9
MVPA Weekday	424	55.9	29.4	680	53.3	25.2	452	72.3	21.4	402	62.4	17.8	854	67.6	20.4
MVPA Weekend	419	45.6	27.8	627	42.7	35.5	415	70.7	29.8	370	61.9	23.6	785	66.6	27.4
PA guideline**	n	Met (%)	Not met (%)	n	Met (%)	Not met (%)	n	Met (%)	Not met (%)	n	Met (%)	Not met (%)	n	Met (%)	Not met (%)
Overall	423	85.1	14.9	651	80.2	19.8	436	70.9	29.1	386	53.1	46.9	822	62.5	37.5
Weekday	424	84.2	15.8	680	83.2	16.8	452	69.9	30.1	402	52.7	47.3	854	61.8	38.2
Weekend	419	65.4	34.6	627	61.4	38.6	415	60.0	40.0	370	51.4	48.7	785	55.9	44.1

**Current UK recommendations for children: 60 mins MVPA per day; for adults: 30 mins MVPA per day; % ages met and not met.

There were weak positive associations between parental and children’s MVPA which were not markedly altered by adjustment for parental age and BMI, and IMD (Table 3). These multivariable regression results indicate that the magnitude of association of fathers’ time spent in MVPA was similar in sons and daughters, and that for mothers the association appeared stronger for daughters than it did for sons (0.16 minutes per minute increase of time in MVPA of the mother, compared with 0.05 minutes for sons). There was, however, no evidence that the association of mothers’ PA with daughters’ differed from that of mothers’ PA with sons’.

**Table 3:** Linear regression of children’s MVPA predicted by parental MVPA

	**All children**		**Sons**		**Daughters**		
	**N**	**Beta [95%CI]**^**$**^	**N**	**Beta [95%CI]**^**$**^	**N**	**Beta [95%CI]**^**$**^	P for heterogeneity*
Father: Model 1	423	0.09 [−0.01 to 0.19]	227	0.10 [−0.02 to 0.22]	196	0.07 [−0.04 to 0.19]	0.757
Father: Model 2	423	0.09 [−0.01 to 0.18]	227	0.09 [−0.03 to 0.21]	196	0.07 [−0.04 to 0.18]	0.764
Mother: Model 1	651	0.09 [0.03 to 0.17]	337	0.06 [−0.06 to 0.18]	314	0.15 [0.07 to 0.24]	0.188
Mother: Model 2	651	0.10 [0.02 to 0.17]	337	0.05 [−0.07 to 0.17]	314	0.16 [0.07 to 0.25]	0.173

^$^Beta coefficient for child MVPA minutes per minute of parent MVPA.

*Testing that associations are different between time spent in MVPA by daughters and by sons; tested by adding an interaction term (parent MVPA*child gender) into the regression model.

Model 1: Unadjusted association.

Model 2: Adjusted for parent’s age, parent’s BMI, and household IMD.

When these analyses were repeated using dichotomised versions of both parental and child MVPA representing whether recommended levels of PA had been met, children whose fathers met adult levels were 84% more likely to meet child guidelines. Associations were similar between mothers and both genders of children; sons were 68% more likely, and daughters were 63% more likely to meet recommended levels of MVPA if their mother had done so (Table 4).

**Table 4:** Logistic regression predicting whether children meet the recommended levels for PA based on parental PA

	**All children**		**Sons**		**Daughters**		
	**N**	**OR for child meeting recommendations for PA if parent meets them [95% CI]**	**N**	**OR for child meeting recommendations for PA if parent meets them [95% CI]**	**N**	**OR for child meeting recommendations for PA if parent meets them [95% CI]**	P for heterogeneity*
Father: Model 1	423	1.90 [1.02 to 3.52]	227	2.00 [0.85 to 4.68]	196	1.52 [0.64 to 3.60]	0.414
Father: Model 2	423	1.84 [1.00 to 3.39]	227	2.00 [0.87 to 4.61]	196	1.41 [0.60 to 3.28]	0.660
Mother: Model 1	651	1.63 [1.09 to 2.43]	337	1.68 [1.10 to 2.56]	314	1.62 [0.86 to 3.05]	0.922
Mother: Model 2	651	1.62 [1.09 to 2.41]	337	1.68 [1.10 to 2.55]	314	1.63 [0.86 to 3.08]	0.919

*Testing that associations are different between time spent in MVPA by daughters and by sons; tested by adding an interaction term (parent MVPA*gender) into the regression model.

Model 1: Unadjusted association.

Model 2: Adjusted for parent’s age, parent’s BMI, and household IMD.

Weekday MVPA patterns were comparable to overall MVPA. On weekdays children were 50% more likely to meet PA recommendations if their mothers met the adult recommendation compared with children whose mothers failed to achieve this level (OR = 1.50, 95% CI = 1.03 to 2.17, p = 0.032). Results from linear models suggested that associations between parent and child MVPA for just weekend days the patterns were broadly comparable to the overall models. At weekends children whose mothers met PA guidelines were 33% more likely to have done at least 60 minutes MVPA per day compared with children whose mothers did not reach this recommended level (OR = 1.33, 95% CI = 1.01 to 1.76), (see Additional file 1: Tables A-D).

## Discussion

The data presented in this paper show that 29% of boys and 47% of girls aged five to six years did not meet PA guidelines of 60 minutes per day, and that parental time spent in MVPA was weakly associated with their children’s time spent in MVPA. The magnitude of association indicated that each ten additional minutes of MVPA done by the mother was associated with just one additional minute of MVPA for the child, with similar associations for fathers. The weakness of these associations was not evident when dichotomous variables models were used; these analyses indicated that children were 84% and 62% more likely to meet PA guidelines if the father or mother, respectively, met the adult guidelines. There was no evidence of a child gender difference in the association between the PA patterns of parents and children, suggesting that the associations with parental PA were similar for boys and girls. Supplementary analyses indicated that associations were similar when time spent in MVPA on weekday and weekend days were examined separately.

The major strength of this study is the provision of objective physical activity data from both children and their parents at the start of primary school. The study is limited by the absence of both maternal and paternal data for all children, the availability of which would have facilitated a more detailed examination of differences between the associations of mothers’ and fathers’ MVPA with that of their children. It is also important to highlight that in this study 80% of mothers and 85% of fathers met physical activity guidelines. This is markedly higher than then 66% of men and 56% of women who met the PA guidelines in the 2012 Health Survey for England based on self-reported estimates of PA [24]. While the greater higher levels of compliance with the guideline in our sample can be partially attributed to differences in sample age (our sample was limited to parents of young children), the large disagreement in guideline compliance suggests that we recruited a relatively active sample of adults.

Among older children there has been some evidence of an association between parent and child PA when self-report measures were used. For example, results from the ENERGY project showed associations between the self-reported PA of parents and their 10 to 12 year old children in five of seven EU countries for girls, and four of seven countries for boys. It is important to note however, that the coefficients for parental MVPA ranged from −0.028 to 0.195 for girls and −0.044 to 0.226 for boys suggesting that the magnitude of the associations was reasonably comparable to the associations reported here [25]. Previous studies that have used accelerometers in children and parents have yielded mixed results. In a small sample of 80, three- to five-year old Hispanic children there was a strong correlation (r = 0.739, p < 0.001) between accelerometer assessed minutes of moderate intensity PA of children and their mothers [26]. The markedly stronger associations in this study may indicate that among pre-school children there is a stronger relationship between parental and child MVPA.

In our own research using accelerometers we found no evidence of an association between the PA of 10 to 11 year old children and their parents [11]. In contrast, a Canadian pedometer study with five- to 19 year old children reported that every 1000 step increase in fathers’ pedometer steps was associated with 407 extra steps for boys and 273 steps for girls [27]. The stronger associations evident in this study may be a function of measurement device; pedometers capture walking well but are not as good as accelerometers at capturing more intermittent, higher intensity PA [28]. Collectively, these studies have provided a mixed picture which has suggested that patterns of association may differ by age and method of assessing PA. The results of the current study advance current knowledge by providing information on accelerometer assessed PA of a large sample of parents and their young children.

Our results highlight a clear need to develop new approaches to increase the MVPA levels of the 29% of boys and 47% of girls who did not meet PA guidelines. Since PA levels decline with age [6], compliance with PA guidelines is only likely to decrease as children age indicating that approaches to increase child PA and prevent the age-related decline are needed. Previous research has shown that PA parenting practices (the specific actions that a parent takes to hinder or facilitate PA for his or her child) are associated with greater accelerometer assessed PA among older children and adolescents [29-32]. It is not unreasonable to assume that similar effects will be evident for younger children and efforts to promote child PA should focus on how parents can facilitate PA for their child. The key message is that the parents of young children should strive to facilitate increased PA amongst their children.

Using the binary measures of meeting recommended levels of PA indicated relatively strong associations between parent and child PA with children 84% more likely to meet the PA guidance if their father also met the guidance. Similarly, sons were 68% and daughters were 63% more likely to meet recommended levels of MVPA if their mother had done so. Without the data from the linear regression models these findings would appear to indicate a strong association between parent and child PA. However, the linear models show that these binary associations are largely driven by small differences around the cut-points for classification of meeting or not meeting PA recommendations.

The results presented here markedly improve current knowledge about the associations between the physical activity patterns of children and their parents, but there are two important issues that still need to be addressed: 1) it is not clear how these associations change as children age, and if associations between parents and children vary with the age of the child; 2) although the research advances the current literature by demonstrating the existence of only a weak association between parent and child PA patterns, the current data cannot provide insight into how best to work with parents to change the PA behaviour of young children. This last point is critical as the data presented here suggest a need to develop new strategies to increase child PA and ensure that more children engage in adequate amounts of PA. It is also important to highlight that the current analyses has focussed solely on the direct associations between the physical activity of children and their parents and has not provided information about parental modelling of activity or the extent to which parents facilitate activity for their children. Studies that examine these issues among young children are required.

## Conclusions

In this study we have shown that 29% of boys and 47% of girls aged five to six years did not meet PA guidelines. Parental time spent in MVPA was very weakly associated with children’s MVPA. Health professionals should encourage parents to focus on creating activity opportunities for their children.

## Competing interests

The authors declare that they have no competing interest.

## Authors’ contributions

RJ, SJS, JLT and DAL were involved in the design of this study and in seeking funding for it. RJ, LP and JZ were responsible for the study conduct with LP managing data collection. LW performed all analyses. RJ wrote the first draft of the paper and coordinated contributions from other co-authors. All authors made critical comments on drafts of the paper. All authors read and approved the final manuscript.

## Pre-publication history

The pre-publication history for this paper can be accessed here:

http://www.biomedcentral.com/1471-2458/14/655/prepub

## Supplementary Material

Additional file 1: Table A Linear regression of children’s MVPA predicted by parental MVPA (weekdays). **Table B.** Logistic regression predicting whether children meet the recommended levels for PA based on parental PA (weekdays). **Table C.** Linear regression of children’s MVPA predicted by parental MVPA (weekdays). **Table D.** Logistic regression predicting whether children meet the recommended levels for PA based on parental PA (weekend days). Click here for file
