# Supplementary material for: Associations between objectively assessed child and parental physical activity: a cross-sectional study of families with 5–6 year old children
Source: BMC Public Health. 2014 Jun 27;14:655. doi: 10.1186/1471-2458-14-655 (PMC4091740; doi:10.1186/1471-2458-14-655)
Supplement: Additional file 1: Table A — Linear regression of children’s MVPA predicted by parental MVPA (weekdays). Table B. Logistic regression predicting whether children meet the recommended levels for PA based on parental PA (weekdays). Table C. Linear regression of children’s MVPA predicted by parental MVPA (weekdays). Table D. Logistic regression predicting whether children meet the recommended levels for PA based on parental PA (weekend days). [file 1471-2458-14-655-S1.docx]

Table A: Linear regression of children’s MVPA predicted by parental MVPA for weekdays (Bristol 2012)

|  | All children | | Sons | | Daughters | |  |
| --- | --- | --- | --- | --- | --- | --- | --- |
|  | N | Mean difference in child’s time  spent in MVPA per minute of parent time in MVPA (mins) [95%CI] | N | Mean difference in child’s time  spent in MVPA per minute of parent time in MVPA (mins) [95%CI] | N | Mean difference in child’s time  spent in MVPA per minute of parent time in MVPA (mins) [95%CI] | P for heterogeneity* |
| Male parent : Model 1 | 424 | 0.05 [-0.02 to 0.13] | 225 | 0.04 [-0.07 to 0.15] | 199 | 0.07 [-0.03 to 0.16] | 0.738 |
| Male parent : Model 2 | 424 | 0.05 [-0.02 to 0.13] | 225 | 0.03 [-0.09 to 0.14] | 199 | 0.06 [-0.03 to 0.16] | 0.715 |
| Female parent : Model 1 | 680 | 0.05 [-0.02 to 0.11] | 353 | 0.03 [-0.07 to 0.12] | 327 | 0.09 [0.01 to 0.16] | 0.302 |
| Female parent : Model 2 | 680 | 0.06 [-0.01 to 0.12] | 353 | 0.03 [-0.07 to 0.14] | 327 | 0.06 [0.02 to 0.17] | 0.273 |

*Testing that associations are different between time spent in MVPA by daughters and by sons; tested by adding an interaction term (parent MVPA*child gender) into the regression model

Model 1: Unadjusted association

Model 1: Unadjusted association

Model 2: Adjusted for parent’s age, parent’s BMI, and household IMD

Table B: Logistic regression predicting whether children meet the recommended levels for PA based on parental PA for weekdays (Bristol 2012)

|  | All children | | Sons | | Daughters | |  |
| --- | --- | --- | --- | --- | --- | --- | --- |
|  | N | OR for child meeting recommendations for PA if parent meets them [95% CI] | N | OR for child meeting recommendations for PA if parent meets them [95% CI] | N | P for heterogeneity* | P for heterogeneity* |
| Male parent : Model 1 | 424 | 1.52 [0.94 to 2.46] | 225 | 0.96 [0.39 to 2.23] | 199 | 1.69 [0.82 to 3.47] | 0.372 |
| Male parent : Model 2 | 424 | 1.51 [0.93 to 2.46] | 225 | 0.90 [0.37 to 2.18] | 199 | 1.72 [0.84 to 3.52] | 0.377 |
| Female parent : Model 1 | 680 | 1.43 [0.99 to 2.06] | 353 | 1.34 [0.76 to 2.34] | 327 | 1.55 [0.88 to 2.72] | 0.740 |
| Female parent : Model 2 | 680 | 1.50 [1.03 to 2.17] | 353 | 1.38 [0.78 to 2.45] | 327 | 1.64 [0.94 to 2.87] | 0.647 |

*Testing that associations are different between time spent in MVPA by daughters and by sons; tested by adding an interaction term (parent MVPA*child gender) into the regression model

Model 1: Unadjusted association

Model 1: Unadjusted association

Model 2: Adjusted for parent’s age, parent’s BMI, and household IMD

Table C: Linear regression of children’s MVPA predicted by parental MVPA for weekend days (Bristol 2012)

|  | All children | | Sons | | Daughters | |  |
| --- | --- | --- | --- | --- | --- | --- | --- |
|  | N | Mean difference in child’s time  spent in MVPA per minute of parent time in MVPA (mins) [95%CI] | N | Mean difference in child’s time  spent in MVPA per minute of parent time in MVPA (mins) [95%CI] | N | Mean difference in child’s time  spent in MVPA per minute of parent time in MVPA (mins) [95%CI] | P for heterogeneity* |
| Male parent : Model 1 | 419 | 0.20 [0.08 to 0.31] | 226 | 0.21 [0.04 to 0.37] | 193 | 0.16 [0.04 to 0.28] | 0.669 |
| Male parent : Model 2 | 419 | 0.19 [0.08 to 0.29] | 226 | 0.19 [0.04 to 0.35] | 193 | 0.16 [0.02 to 0.29] | 0.669 |
| Female parent : Model 1 | 627 | 0.10 [-0.02 to 0.22] | 323 | 0.05 [-0.06 to 0.16] | 304 | 0.21 [0.12 to 0.31] | 0.028 |
| Female parent : Model 2 | 627 | 0.09 [-0.03 to 0.21] | 323 | 0.04 [-0.07 to 0.15] | 304 | 0.20 [0.10 to 0.30] | 0.025 |

*Testing that associations are different between time spent in MVPA by daughters and by sons; tested by adding an interaction term (parent MVPA*child gender) into the regression model

Model 1: Unadjusted association

Model 1: Unadjusted association

Model 2: Adjusted for parent’s age, parent’s BMI, and household IMD

Table D: Logistic regression predicting whether children meet the recommended levels for PA based on parental PA for weekend days (Bristol 2012)

|  | All children | | Sons | | Daughters | |  |
| --- | --- | --- | --- | --- | --- | --- | --- |
|  | N | OR for child meeting recommendations for PA if parent meets them [95% CI] | N | OR for child meeting recommendations for PA if parent meets them [95% CI] | N | OR for child meeting recommendations for PA if parent meets them [95% CI] | P for heterogeneity* |
| Male parent : Model 1 | 419 | 1.65 [1.04 to 2.61] | 226 | 1.56 [0.86 to 2.85] | 193 | 1.78 [0.96 to 3.30] | 0.748 |
| Male parent : Model 2 | 419 | 1.55 [0.98 to 2.47] | 226 | 1.44 [0.79 to 2.64] | 193 | 1.78 [0.94 to 3.36] | 0.610 |
| Female parent : Model 1 | 627 | 1.32 [1.01 to 1.74] | 323 | 1.69 [1.12 to 2.56] | 304 | 1.10 [0.72 to 1.60] | 0.137 |
| Female parent : Model 2 | 627 | 1.33 [1.01 to 1.76] | 323 | 1.73 [1.14 to 2.62] | 304 | 1.03 [0.94 to 1.58] | 0.140 |

*Testing that associations are different between time spent in MVPA by daughters and by sons; tested by adding an interaction term (parent MVPA*child gender) into the regression model

Model 1: Unadjusted association

Model 1: Unadjusted association

Model 2: Adjusted for parent’s age, parent’s BMI, and household IMD
